# Appropriate usage of food thickening agents to prevent non-disintegration of magnesium oxide tablets

**DOI:** 10.1038/s41598-020-73135-8

**Published:** 2020-09-30

**Authors:** Taisuke Matsuo, Airi Sato, Kenzo Kudo, Yasuyuki Sadzuka, Takashi Tomita

**Affiliations:** 1 Division of Advanced Pharmaceutics, Department of Clinical Pharmaceutical Sciences, School of Pharmacy, Iwate Medical University, 1–1–1 Idaidori, Yahaba-cho, Shiwa-gun, Iwate, 028–3694 Japan; 2 Division of Clinical Pharmaceutics and Pharmacy Practice, Department of Clinical Pharmacy, School of Pharmacy, Iwate Medical University, 1–1–1 Idaidori, Yahaba-cho, Shiwa-gun, Iwate, 028–3694 Japan; 3 Department of Pharmacy, Iwate Medical University Hospital, 2–1–1 Idaidori, Yahaba-cho, Shiwa-gun, Iwate, 028–3694 Japan; 4 Department of Pharmaceutical Sciences, Faculty of Pharmaceutical Sciences, Teikyo Heisei University, 4-21-2, Nakano, Nakano-ku, Tokyo, 164-8530 Japan

**Keywords:** Health care, Risk factors

## Abstract

Food thickening agents are used to aid the administration of medicine to elderly patients with dysphagia. Magnesium oxide tablets are sometimes administered with food thickening agents. Non-disintegration and disintegration delay of these tablets in the body are problems associated with food thickening agent use. However, the appropriate usage of food thickening agents for administering tablets is not established. Here, the reasons for the non-disintegration of magnesium oxide tablets administered with food thickeners and appropriate usage of food thickeners were examined using a disintegration test of newly opened and moisture-absorbed magnesium oxide tablets. Immersion of magnesium oxide tablets for 10 and 30 min in xanthan and guar gum-based food thickening agents caused disintegration delay and non-disintegration in the first fluid (pH 1.2). However, tablets immersed for 1 min quickly disintegrated. The disintegration of xanthan gum-based food thickening agents was faster than guar gum-based food thickening agents. Moisture absorption by magnesium oxide tablets caused a significant delay in their disintegration in water. The tablets that absorbed moisture disintegrated within 1 min in the first fluid, even when immersed in food thickening agents for a short time. Overall, a short immersion of magnesium oxide tablets in food thickening agents can avoid non-disintegration.

## Introduction

Food thickening agents (food thickeners) are auxiliary foods that aid in swallowing. They are used to aid in administering not only food, but also medicines to elderly patients with dysphagia in hospitals and nursing care facilities^1,2^. Food thickeners prevent accidental aspiration by reducing the swallowing speed. Food thickeners are classified into the following three groups: starch-based (first generation), guar gum-based (second generation), and xanthan gum-based thickeners (third generation)^2^. Generally, a patient dissolves the food thickener with a liquid, such as water, to the preferred viscosity. However, food thickeners decrease the pharmacokinetics and activities of several medicines^3–7^, due to a delay in the disintegration time and reduction in the dissolution rate^8^.

Magnesium oxide is frequently used for its antacid and laxative properties in elderly patients. The laxative effect of magnesium oxide tablets occurs via two steps: (i) the tablets disintegrate and release magnesium oxide particles, and (ii) these particles react with gastric acid and form magnesium bicarbonate^5,8^. It is essential that magnesium oxide tablets disintegrate and dissolve in the stomach to exert their effects. Although magnesium oxide tablets rapidly disintegrate in water in seconds, non-disintegrated tablets in the stool of patients who swallowed the tablets with food thickeners have once been reported^9^. However, the reasons for this observation are not clear. Tomita et al. showed that food thickeners of high thickness delay disintegration in water more than those of low thickness^10,11^. The disintegration tests of magnesium oxide tablets are generally performed in water^12,13^. However, the tablets administered with food thickeners generally disintegrate in the stomach. Thus, disintegration test in the first solution (pH 1.2) is desirable. Additionally, elderly people are often prescribed several medicines, and these, including magnesium oxide tablets, are sometimes dispensed as one-dose packages to easily manage drug administration. The disintegration time of magnesium oxide tablets in water is delayed due to moisture absorption^12–14^. A further delay in disintegration may be caused by food thickeners.

In this study, the reasons for the non-disintegration of magnesium oxide tablets administered with food thickeners and the appropriate usage of food thickeners were examined using a disintegration test of newly opened and moisture-absorbed magnesium oxide tablets.

## Results

### International dysphagia diet standardization initiative (IDDSI) flow test of food thickeners

**Figure 1:**
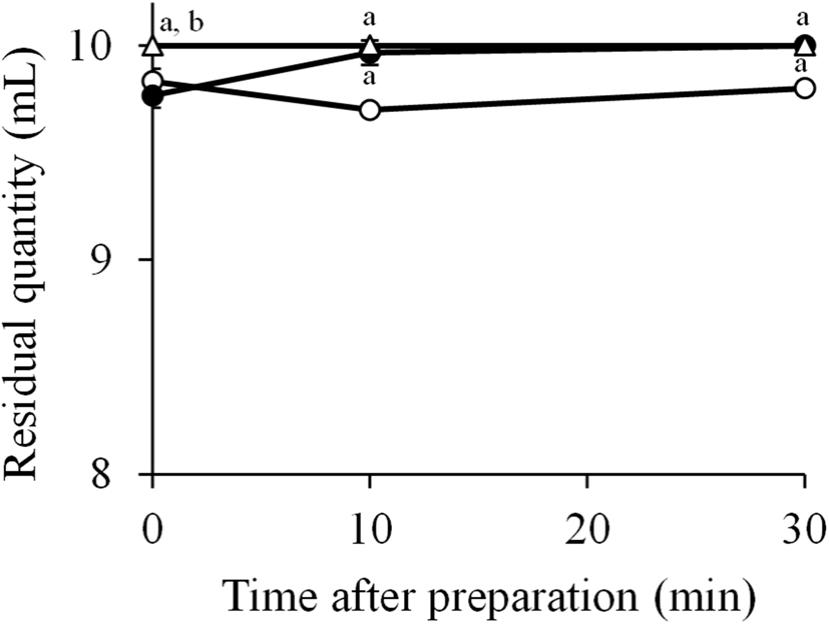
IDDSI test with food thickeners. The IDDSI flow test was performed using three food thickeners at 0, 10, and 30 min after preparation. Open circle: Tsururinko Quickly (3%, w/v), filled circle: Hightoromeal (2.7%, w/v), open triangle: Neo-Hightoromeal III (2.5%, w/v). P < 0.001 represents a significant difference (compared with ^a^Tsururinko Quickly and ^b^Hightoromeal at each time).

The relationship between viscosity and time after the preparation of food thickeners was examined using the IDDSI flow test (Fig. 1). A IDDSI flow test value of xanthan-gum based Tsururinko Quickly and Neo-Hightoromeal III was 9.7–9.8 (moderately thick) and 10.0 (extremely thick) for 30 min after preparation, respectively. The IDDSI flow test value of guar-gum based Hightoromeal increased from 9.8 at 0 min to 10.0 at 30 min.

### Effects of immersion time in food thickeners on the disintegration time of magnesium oxide tablets

The relationship between immersion time in food thickeners and disintegration time of Magmitt tablets was examined. The disintegration time of Magmitt tablets in the first fluid (pH 1.2) was approximately 5 s (Table 1). The average disintegration time of tablets immersed in food thickeners for 30 min was more than 1 h (3600 s) (Table 1). However, in the Tsururinko Quickly, Hightoromeal, and Neo-Hightoromeal III thickeners, two, four, and four tablets did not disintegrate within 2 h, respectively. The disintegration time after 10 min of immersion was lower than that after 30 min of immersion. However, some tablets did not disintegrate. When tablets were immersed for 1 min in xanthan gum-based Tsururinko Quickly and Neo-Hightoromeal III, the average time for disintegration was less than 10 s. When guar gum-based Hightoromeal was used, the average disintegration time was approximately 15 min (900 s). In all cases, all tablets were disintegrated (Table 1).

**Table 1:** Effect of immersion time of magnesium oxide tablets into food thickeners on the disintegration time (Magmitt Tab.)

	Immersion time	Disintegration time (s)		Number of non-disintegrating tablets
		Range	Mean	
Food thickeners	No immersion	4–7	5.1 ± 1.2	0
Tsururinko Quickly	1 min	4–9	5.5 ± 1.7	0
	10 min	12– > 7200	1590.0 ± 2917.1	2
	30 min	1260– > 7200	4109.5 ± 2152.1***	2
Hightoromeal	1 min	113–1520	889.3 ± 532.6	0
	10 min	65– > 7200	2744 ± 2419.3**	1
	30 min	100– > 7200	4145 ± 2781.8***	4
Neo-Hightoromeal III	1 min	4–55	9.8 ± 14.4	0
	10 min	13– > 7200	750.3 ± 2043.5	1
	30 min	300– > 7200	4460.8 ± 2794.8***	4

Disintegration test was performed using first fluid (pH 1.2).

**P < 0.01 and ***P < 0.001 represent significant differences (compared to non-immersing tablet).

### Effects of time from preparation to immersion on disintegration time

We examined whether the cause of disintegration delay was immersion time or time from food thickener preparation to use. Food thickeners were used either immediately, 10 min, or 30 min after food thickener preparation. Magmitt tablets were immersed in the thickeners for 1 min. In the two xanthan gum-based thickeners, all tablets immediately disintegrated in the first fluid, and this was observed in all groups (Table 2). In the guar gum-based thickener, the disintegration time of the tablets was longer when the thickener was used 10 and 30 min later than when the thickener was used immediately (Table 2).

**Table 2:** Effect of time from preparation to use of food thickeners on disintegration time of magnesium oxide tablets (Magmitt Tab.)

	Time from preparation to use to use of food thickeners	Disintegration time (s)		Number of non-disintegrating tablets
		Range	Mean	
Food thickeners	No immersion	4–7	5.1 ± 1.2	0
Tsururinko Quickly	0 min	3–14	5.1 ± 3.0	0
	10 min	4–7	4.8 ± 1.0	0
	30 min	4–8	5.2 ± 1.4	0
Hightoromeal	0 min	30–1510	832.3 ± 538.2**	0
	10 min	181–2060	1270.6 ± 585.8***	0
	30 min	180–2640	1363.4 ± 696.7***	0
Neo-Hightoromeal III	0 min	4–10	5.8 ± 2.0	0
	10 min	4–12	6.2 ± 2.7	0
	30 min	4–9	5.0 ± 1.5	0

Disintegration test was performed using first fluid (pH 1.2).

The immersion time was 1 min.

The disintegration time of “No immersion” was same result in Table 1.

**P < 0.01 and ***P < 0.001 represent significant differences (compared to non-immersing tablet).

### Effects of moisture-absorbed magnesium oxide tablets immersed in food thickeners on disintegration time

**Figure 2:**
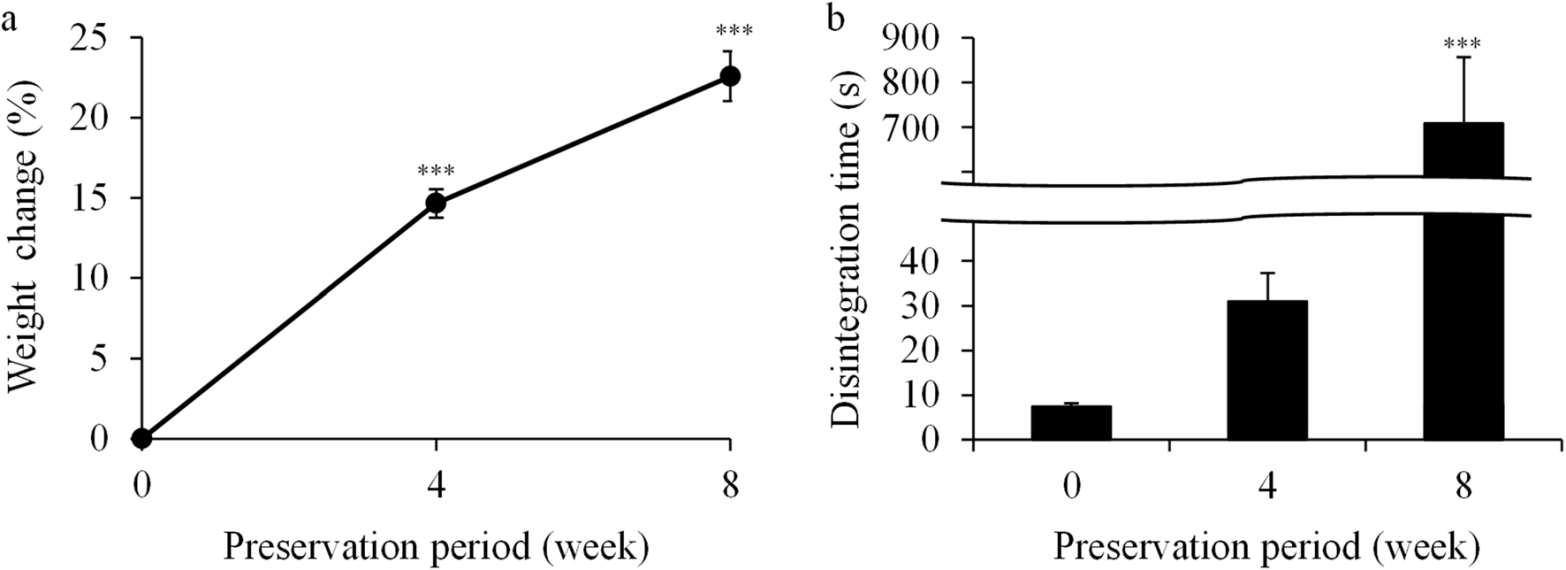
Changes in the characteristics of magnesium oxide tablets stored in a one-dose package. Magmitt Tab. 330 mg in one-dose packages were stored at 25 °C under 75% ± 5% relative humidity for 4 and 8 weeks. **(a)** Weight change of tablets measured after 4 and 8 weeks (n = 50). **(b)** Disintegration time of tablets in water at 37 °C ± 2 °C. ***P < 0.001 represents a significant difference (compared with newly opened tablet (0 week)).

We examined whether moisture-absorbed Magmitt tablets, immersed in food thickeners for 1 min, were disintegrated in the first fluid. The moisture absorption rate of the tablets stored for 4 and 8 weeks was 14.6% and 22.6%, respectively. The disintegration time of the tablets after 4 and 8 weeks of storage was 27 and 709 s in water, respectively, and these were considerably higher than that of newly opened tablets (8 s) (Fig. 2). However, after 4 and 8 weeks of storage, the disintegration time in the first fluid was 11 and 22 s, respectively (Table 3), which were higher than that of newly opened tablets (5 s) (Table 1). The disintegration time in the first fluid was lower than that in water. Furthermore, the stored tablets disintegrated in less than 1 min when they were immersed in each food thickener for 1 min (Table 3).

**Table 3:** Effect of food thickeners on the disintegration time of moisture absorbed magnesium oxide tablets (Magmitt Tab.)

Preservation period	Food thickeners	Disintegration time (s)		Number of non-disintegrating tablets
		Range	Mean	
4 weeks	No immersion	9–11	11.0 ± 1.2	0
	Tsururinko Quickly	9–21	13.2 ± 4.1	0
	Hightoromeal	10–43	23.1 ± 10.5**	0
	Neo-hightoromeal III	13–53	23.9 ± 12.4**	0
8 weeks	No immersion	20–24	21.7 ± 1.5	0
	Tsururinko Quickly	20–48	32.3 ± 7.5	0
	Hightoromeal	28–62	45.1 ± 10.9***	0
	Neo-Hightoromeal III	22–71	37.0 ± 14.5*	0

Disintegration test was performed using first fluid (pH 1.2).

The immersion time is 1 min.

*P < 0.05, **P < 0.01, and ***P < 0.001 represent significant differences (compared to each non-immersing tablet).

### Effects of food thickeners on the disintegration time of other magnesium oxide tablets

**Figure 3:**
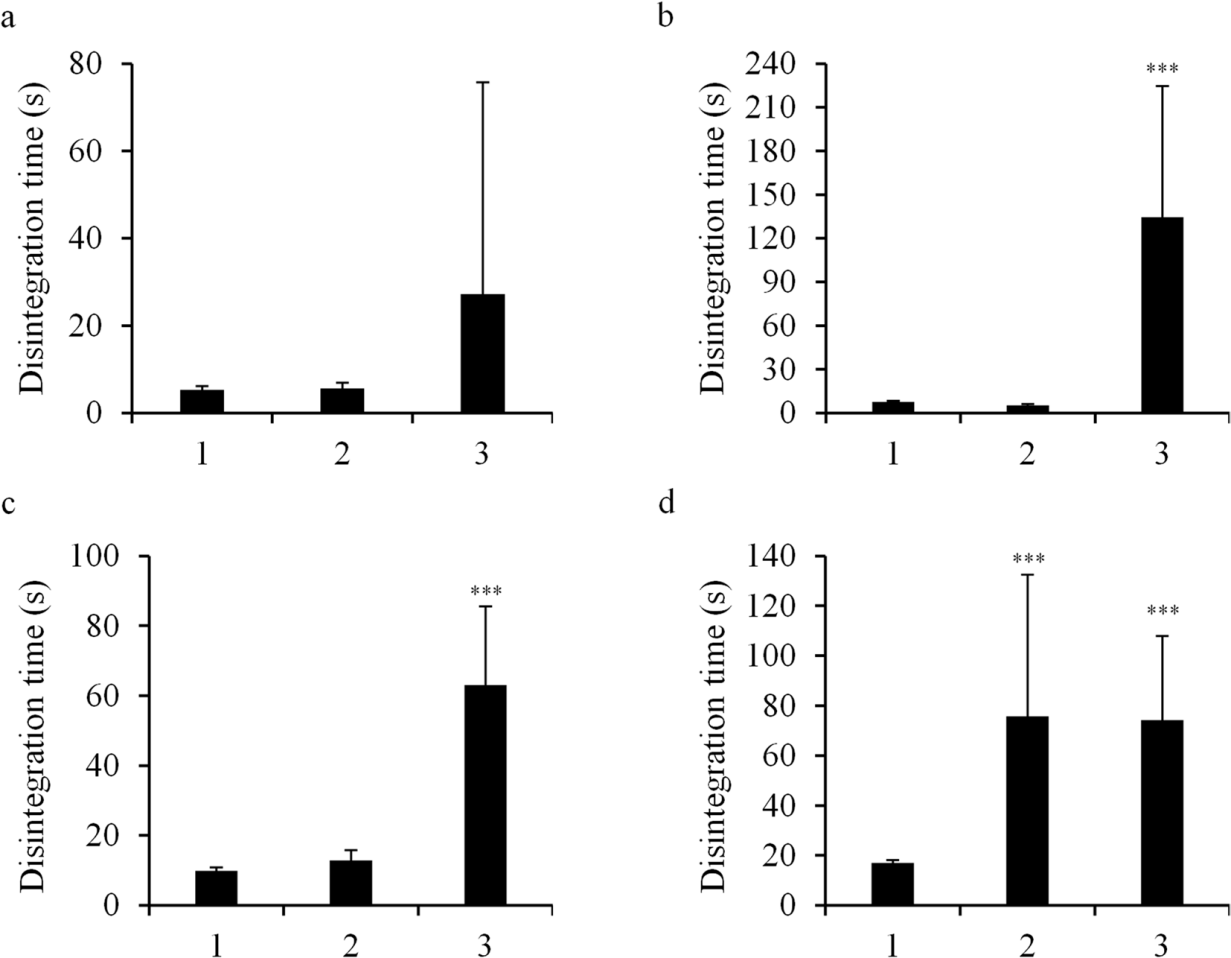
Disintegration time of other magnesium oxide tablets (330 mg) immersed in food thickeners. Magnesium oxide tablets were immersed in food thickeners for 1 min. Disintegration test was performed in the first fluid (pH 1.2). Magnesium oxide (330 mg) “MOCHIDA” **(a)**, “KENEI” **(b)**, “Yoshida” **(c)**, and “Mylan” **(d)**. 1: no immersion, 2: immersed in 3% Tsururinko Quickly, 3: immersed in 2.7% Hightoromeal. ***P < 0.001 represent significant differences (compared with no immersion).

The effects of food thickeners on the disintegration time of other magnesium oxide tablets were examined. The tablets were immersed in the xanthan-gum based Tsururinko Quickly and guar gum-based Hightoromeal, which have similar IDDSI values. Magnesium oxide Tablets “MOCHIDA”, “KENEI”, and “Yoshida” were disintegrated in 5–10 s without food thickeners, similar to Magmitt tablet (Fig. 3). Disintegration delay of these tablets in the guar gum-based food thickener Hightoromeal was longer than that in the xanthan gum-based food thickener Tsururinko Quickly. Disintegration delay of the magnesium oxide tablet “Mylan,” the disintegration time of which without food thickeners was longer than that of other tablets, in both food thickeners was similar (Fig. 3).

### Comparison of adhesiveness between xanthan gum- and guar gum-based food thickeners

**Figure 4:**
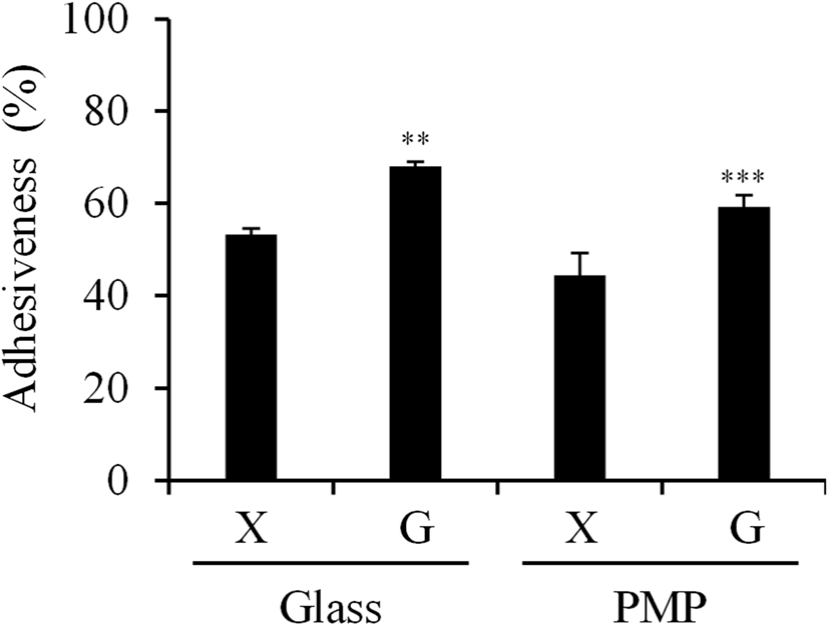
Adhesiveness of xanthan gum- and guar gum-based food thickeners. Xanthan gum-based Tsururinko Quickly (3%, X) and guar gum-based Hightoromeal (2.7%, G) were used in the analyses. Glass or PMP beakers were used. **P < 0.01 and ***P < 0.001 represent significant differences (compared with 3% Tsururinko Quickly).

Adhesiveness between xanthan gum-based Tsururinko Quickly and guar gum-based Hightoromeal was compared based on the adhesion of food thickeners to beakers. In glass and polymethylpentene (PMP) beakers, the adhesiveness of guar gum-based Hightoromeal was approximately 15% was higher than that of xanthan gum-based Tsururinko Quickly (Fig. 4).

## Discussion

A questionnaire survey conducted in care facilities showed that the immersion time of medicines in food thickeners was different in each facility and ranged from immediately to 30 min^15^. In this study, some Magmitt tablets immersed in food thickeners for 10 and 30 min did not disintegrate in the first fluid (pH 1.2) within 120 min (Table 1). The intragastric retention time of medicines is within 120 min^16^; thus, a long immersion time may be a cause of the non-disintegration of magnesium oxide tablets in the stomach.

In the present study, Magmitt tablets (200, 250, and 500 mg) quickly disintegrated when immersed into xanthan gum-based food thickeners for 1 min, but some tablets immersed in guar gum-based food thickeners did not disintegrate within 30 min (data not shown). The effects of food thickeners on the disintegration rate of these tablets were similar to those of Magmitt Tabs 330 mg. The results showed that xanthan gum-based food thickeners were associated with shorter disintegration times; thus, xanthan gum-based thickeners may be the most appropriate for the administration of magnesium oxide tablets. Currently, xanthan gum-based food thickeners are frequently used because of their negligible effects on flavor; moreover, their viscosity can be adjusted quickly^7^. The viscosity of xanthan gum-based food thickeners is more stable after preparation than guar gum- and starch-based food thickeners^2^. Here, the IDDSI values of xanthan gum-based thickeners were stable 30 min after preparation. Thus, xanthan gum-based food thickeners can be prepared ahead in nursing facilities.

The delay in the disintegration of tablets by food thickeners can be attributed to the adhesion of food thickeners to the surface of tablets and their invasion into the voids of tablets. The adhesiveness of guar gum-based Hightoromeal was higher than that of xanthan gum-based Tsururinko Quickly although their IDDSI values were similar. Therefore, the disintegration time of the majority of magnesium oxide tablets immersed in guar gum-based Hightoromeal was higher than that of tablets immersed in xanthan gum-based food thickeners. The main chain of the xanthan gum molecule is glucose (β-1,4-glycosidic bond). It has many side chains and a large negative charge derived from the carboxyl group and pyruvate^17^. Regarding the viscosity of the xanthan gum solution, the cation between xanthan gum molecules is important for their aggregation. On the contrary, guar gum is a neutral galactomannan^18^. This difference may cause differences in their adhesiveness and effects on the disintegration of tablets. Additionally, the disintegration time of the moisture-absorbed magnesium oxide tablets in Hightoromeal was shorter than that of newly opened tablets in Hightoromeal. It has been reported that voids in magnesium oxide tablets are decreased when the tablets absorb moisture^19,20^. This was because, although Hightoromeal easily invades newly opened tablets, the invasion of thickener into moisture-absorbed tablets may be difficult. To clarify the mechanism underlying disintegration delay of the magnesium oxide tablets immersed in food thickeners, it is crucial to directly observe the invasion of food thickeners into the tablets by noninvasive monitoring using X-ray computed tomography analysis.

The dose of magnesium oxide tablets is higher for people who use food thickeners than that for people who do not use food thickeners^8^. In a previous study, it was demonstrated that the dissolution rate of magnesium oxide tablets immersed in 3% Tsururinko Quickly and 2.7% Hightoromeal for 30 min was 26% and 3%, respectively, whereas that of the tablets not immersed in food thickeners for 120 min was over 80%^8^. Our results showed that the disintegration time of magnesium oxide tablets immersed in food thickeners for 30 min was significantly delayed (Table 1), and this delay may be the cause of the reduction in the dissolution rate. Although all magnesium oxide tablets used in this study were uncoated tablets, the characteristics of different tablets are different, even when the main component is the same. Medicines for oral administration include uncoated tablets, coated tablets, orally disintegrating tablets, and capsules; thus, the effect of food thickeners on each medicine may be different. Further experiments using different types of tablets with food thickeners are necessary.

In conclusion, we demonstrated that a short immersion time of magnesium oxide tablets in food thickeners is important for disintegration. It is desirable to shorten the immersion time of medicines in food thickeners in facilities where the immersion time is long. However, care must be taken to avoid adverse effects caused by the enhanced effects of fast disintegrating medicines. The results of this study will help enhance the effects of orally administered medicines with food thickeners by reducing the disintegration time of medicines in the gastric fluid. Furthermore, the findings can be used to determine the appropriate administration of medicines with food thickeners.

## Materials and methods

### Materials

The following three commercial food thickeners were used: Tsururinko Quickly (xanthan gum based; Clinico Co., Ltd., Japan, 3.0 g/pack), Hightoromeal (guar gum based; Foodcare Inc., Japan, 2.7 g/pack), and Neo-Hightoromeal III (xanthan gum based; Foodcare Inc., 2.5 g/pack). The following five magnesium oxide tablets were used: Magmitt Tab. 330 mg (Kyowa Chemical Industry Co., Ltd., Japan; 19B813), Magnesium Oxide Tablet 330 mg “Mylan” (Mylan Inc., USA; M220AB5), Magnesium Oxide Tablet 330 mg “MOCHIDA” (Mochida Pharmaceutical Co., Ltd., Japan; B749), Magnesium Oxide Tablet 330 mg “Yoshida” (Yoshida Pharmaceutical Co., Ltd., Japan; 919105), and Magnesium Oxide Tablet 330 mg “KENEI” (Kenei Pharmaceutical Co., Ltd., Japan; 9A05).

### Preparation of food thickener solutions

The food thickeners were dissolved in 100 mL of soft water (natural mineral water from the South Japanese Alps; Suntory Beverage & Food Limited, Japan). The concentration of Tsururinko Quickly, Hightoromeal, and Neo-Hightoromeal III was 3% (w/v), 2.7% (w/v), and 2.5% (w/v), respectively. The dissolved food thickeners were allowed to stand for 2 min after mixing and dissolution (this condition was defined as after the preparation and before use in the experiments).

### IDDSI flow test

The IDDSI flow test was performed using a 10-mL syringe according to the IDDSI method. After removing the plunger, the nozzle of the syringe was covered with a finger, and then 10 mL of food thickeners was added. The nozzle was released for 10 s, and then the residual quantity was measured. The food thickeners were classified as follows: extremely thick (level 4; 10 mL), moderately thick (level 3; 8–10 mL), mildly thick (level 2; 4–8 mL), slightly thick (level 1; 1–4 mL), and thin (level 0; 0 mL).

### Immersion of magnesium oxide tablets in food thickeners

To examine the relationship between immersion time and disintegration time of magnesium oxide tablets, the tablets were immersed in food thickeners for 1, 10, and 30 min. On the contrary, to clarify effects of the time until use of food thickeners after their preparation, the food thickeners were used after 0, 10, and 30 min of preparation. The immersion time was 1 min. In both experiments, after immersion, the tablets were placed in the container baskets of a disintegration tester using a dispensing spoon and the disintegration test was performed as described in the “Disintegration test” below.

### Storage of magnesium oxide tablets in a one-dose package

Magmitt Tab. 330 mg was used. Two tablets of Magmitt Tab. 330 mg were packaged in cellophane polyethylene laminate. The tablets in one-dose packages were stored at 25 °C under 75% ± 5% relative humidity for 4 and 8 weeks. After weighing the tablets using an electronic analytical balance, the tablets were used for analysis as described in the “Immersion of magnesium oxide tablets into food thickeners.”

### Disintegration test

The disintegration test was performed according to the disintegration test method of the Japanese Pharmacopoeia (17th Edition) using a disintegration tester (NT-40HS, Toyama Sangyo Co., Ltd., Japan). Test solutions were the first fluid (sodium chloride (2.0 g) and hydrochloric acid (7.0 mL) in 1000 mL of water, pH 1.2) and water at 37 °C ± 2 °C. The rotation speed was 30 rpm. Each test was performed with 12 tablets. For the tablet disintegration test after immersion in food thickeners, the food thickeners could not be completely removed from the surface of the tablets, that is, there were residual food thickeners on the tablets. Therefore, the time when the contents of the tablets were released was defined as the disintegration time of tablets. When the tablets were not disintegrated within 2 h, the tablets were defined as non-disintegratable.

### Adhesiveness of food thickeners

We added 10 g (A) of 3.0% xanthan gum-based Tsururinko Quickly and 2.7% guar-gum based Hightoromeal into 100 mL glass or PMP beakers. We then placed the beakers upside-down on pre-weighed plastic dishes (B) for 1 min. Subsequently, the plastic dishes with food thickeners were weighed (C). Adhesiveness of food thickeners was calculated using the following formula.

$$ {\text{Adhesiveness}}\;\left( \% \right) = \left[ {\left( {\text{A}} \right) - \left( {\left( {\text{C}} \right) - \left( {\text{B}} \right)} \right)} \right]/\left( {\text{A}} \right) \times 100 $$

### Statistical analysis

The data are presented as mean ± SD. Statistical analyses were performed using a one-way ANOVA (post-hoc test: Dunnett’s test) or *t*-test in BellCurve for Excel, version 3.20 (Social Survey Research Information Co., Ltd., Japan). A p value of < 0.05 indicated a statistically significant difference.
